# Looking at Social Interactions in Medical Education with Dual Eye-Tracking Technology: A Scoping Review

**DOI:** 10.12688/mep.20577.1

**Published:** 2024-10-10

**Authors:** Johannes Lorenz, Juliane Zevano, Nils Otto, Bertrand Schneider, Cihan Papan, Markus Missler, Dogus Darici

**Affiliations:** 1 University of Münster, Münster, Germany; 2 Institute for Anatomy and Neurobiology, University of Münster, Münster, Germany; 3 Harvard Graduate School of Education, Cambridge, Massachusetts, USA; 4 Insistute for Hygiene and Public Health, University Hospital Bonn, Bonn, Germany

**Keywords:** Eye-Tracking, Dual Eye-Tracking, Dual mobile Eye-Tracking, Scoping Review, Medical Education

## Abstract

**Purpose:**

Social interactions are fundamental to effective medical practice, yet assessing these complex dynamics in educational settings remains challenging. This review critically examines the emerging use of dual eye-tracking technology as a novel method to quantify, analyze, and enhance social interactions within medical education contexts.

**Materials and Methods:**

We performed a scoping review of the literature, focusing on studies that utilized dual eye-tracking within medical education contexts. Our search included multiple databases and journals. We extracted information on technical setups, areas of application, participant characteristics, dual eye-tracking metrics, and main findings.

**Results:**

Ten studies published between 2012 and 2021 met the inclusion criteria, with 90% utilizing dual screen-based- and 10% dual mobile eye-tracking. All studies were conducted in the context of surgical training, primarily focusing on laparoscopic surgery. We identified two main applications of dual eye-tracking: (1) as an educational *intervention* to improve collaboration, (2) as a diagnostic tool to identify interaction pattern that were associated with learning. Key metrics included joint visual attention, gaze delay and joint mental effort.

**Conclusion:**

Dual eye-tracking offers a promising technology for enhancing medical education by providing high-resolution, real-time data on social interactions. However, current research is limited by small sample sizes, outdated technology, and a narrow focus on surgical contexts. We discuss the broader implications and potential for medical education research and practice.

## Practice points

1) Dual eye-tracking provides high-resolution data of social interactions in medical education.
2) It improves training outcomes by visualizing gaze patterns and diagnosing teamwork.
3) Research is currently limited to small studies and outdated technology.
4) Future studies have the potential to expand beyond surgery and employ mobile eye-tracking setups.

## Introduction

Social interactions play a pivotal role in shaping the professional identity, competence, and empathy of future healthcare professionals. From problem-based learning (PBL) that leverages group discussions for solving problems in clinical scenarios, to the apprenticeship model that underscores learning in the context of professional relationships, social interactions form the foundation of effective learning environments in medical education ( Keren *et al.*, 2017; Kirschner *et al.*, 2009; van den Bossche *et al.*, 2011).

However, capturing and assessing social interactions remains an unsolved problem. Traditional methods, such as self-reporting and observer-based assessments, often fall short in providing comprehensive and objective data on the quality and impact of these interactions ( Groh *et al.*, 2010).

**Figure 1.**
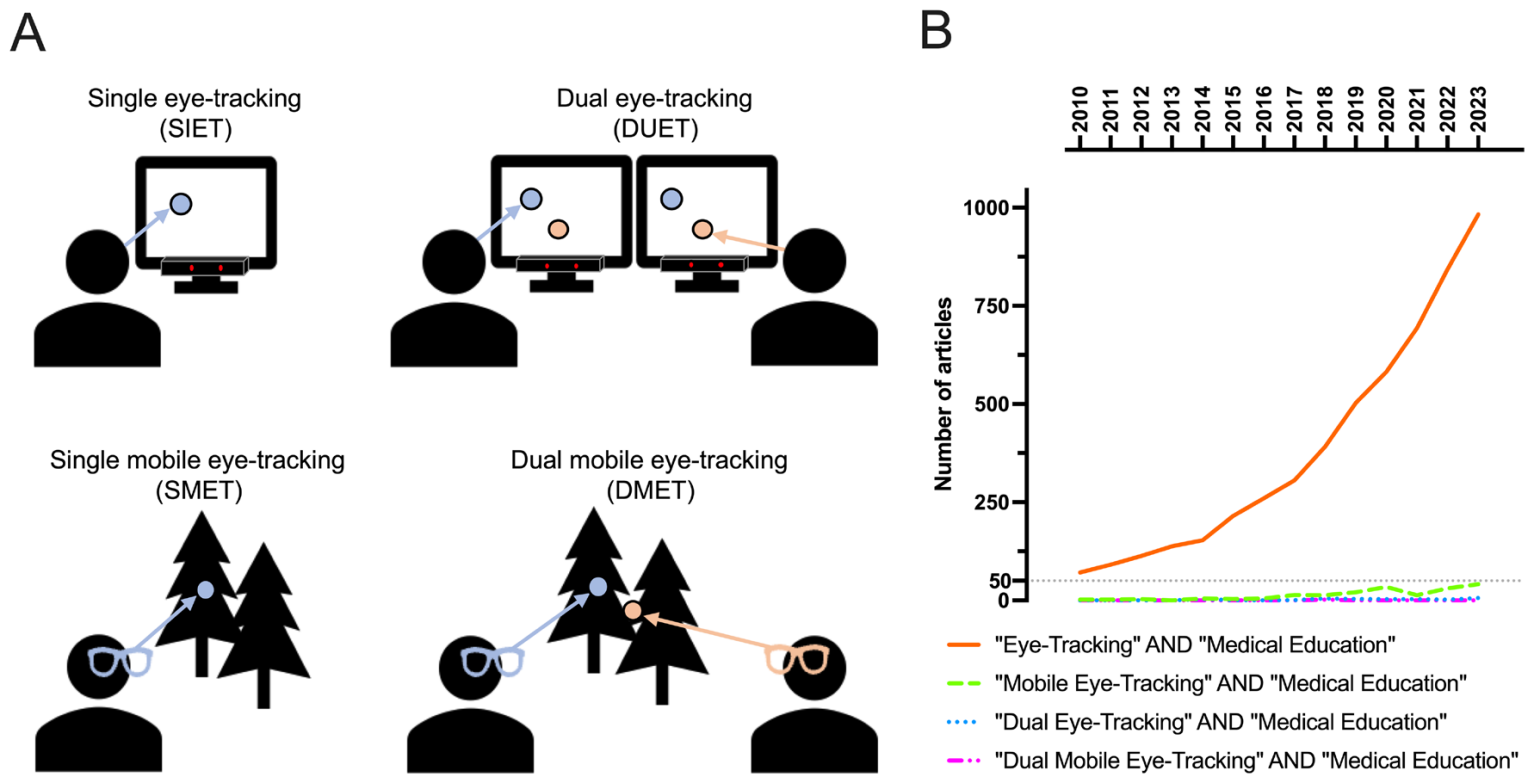
Prevalence of eye-tracking method terminology in medical education increases exponentially. **A**) Different eye-tracking methods used in medical education studies based on two key dimensions: the number of participants (single vs. dual) and the type of eye-tracking equipment used (screen-based vs. mobile). **B**) Quantification of eye-tracking variant use within medical education research from 2010 to 2023. The general term “Eye-Tracking” has become exponentially more used in combination with “Medical Education”. Mobile Eye Tracking has become slightly more prevalent but numbers remain low. Dual eye-tracking have been used a few times only recently.

One approach to address these limitations is dual eye-tracking. This sensor-based technology collects attentional data from two or more participants. Unlike traditional eye-tracking methods, which record the gaze of only one person at a time (single eye-tracking, SIET), dual eye-tracking tracks the eye movements of two individuals simultaneously. This will be done either screen-based (DUET) or mobile (DMET) ( Figure 1, Panel A).

Eye tracking reveals *where* individuals look at, *when* they shift their gaze, and *how long* they fixate on specific areas of interest – which can be used as indicators of attention and underlying cognitive processing ( Brunyé *et al.*, 2019; Darici *et al.*, 2023b). Modern eye-trackers incorporate pupillometry, which involves recording momentary changes in pupil diameter that serves as a psychophysiological index of cognitive load ( Mitre-Hernandez *et al.*, 2021), arousal levels ( Wang *et al.*, 2018), and emotional states ( Chang *et al.*, 2024).

Dual eye-tracking approaches extend these capabilities by providing indicators of the convergence or divergence of two individuals' gaze patterns in real-time. This offers new ways to study dyad-level interaction patterns, such as joint visual attention ( Schneider & Bryant, 2024), joint mental effort ( Sharma & Olsen, 2022), and leader-follower dynamics ( Schneider *et al.*, 2018). These eye-tracking metrics shed light into differential social roles, culturally evolved social routines, and social scripts ( Fusaroli *et al.*, 2014). Importantly, dual eye-tracking metrics have been linked with learning outcomes in higher education, such as problem-solving skills, communication, and collaboration efficiency ( Schneider *et al.*, 2024). Therefore dual eye-tracking holds potential for studying social interactions in medical training, where situational and social awareness, and the ability to read subtle cues are vital to prevent medical errors ( Walshe *et al.*, 2019).

Despite the promising opportunities this technology offers, its application in medical education has been sparse. This stands in contrast to the increasing number of research utilizing SIET ( Figure 1B). An initial screening of the Google Scholar database from 2010 to 2023 revealed a significant rise in publications using the keywords “eye-tracking” and “medical education”, with up to 1,000 new publications in the year 2023. Several review articles on SIET have been well received by the medical education community ( Ashraf *et al.*, 2018; Brunyé *et al.*, 2019; Junior *et al.*, 2024). In contrast, publications using the keywords “dual eye-tracking”, or “dual mobile eye-tracking” were far less common, and no review articles in medical education exist so far.

Given the rising popularity of dual eye-tracking methodology in higher education (e.g., Schneider *et al.*, 2024) and its scarcity in medical education, we decided to conduct a scoping review to map the existing literature, identify key areas of application, and determine the extent and types of knowledge available.

Specifically, we are interested in the following research questions: How many studies have used dual eye-tracking in medical education? What populations have been studied? In which areas of medical education has it been applied so far? What research questions have been addressed and which new eye-tracking metrics emerge from dual eye-tracking?

## Methods

**Figure 2.**
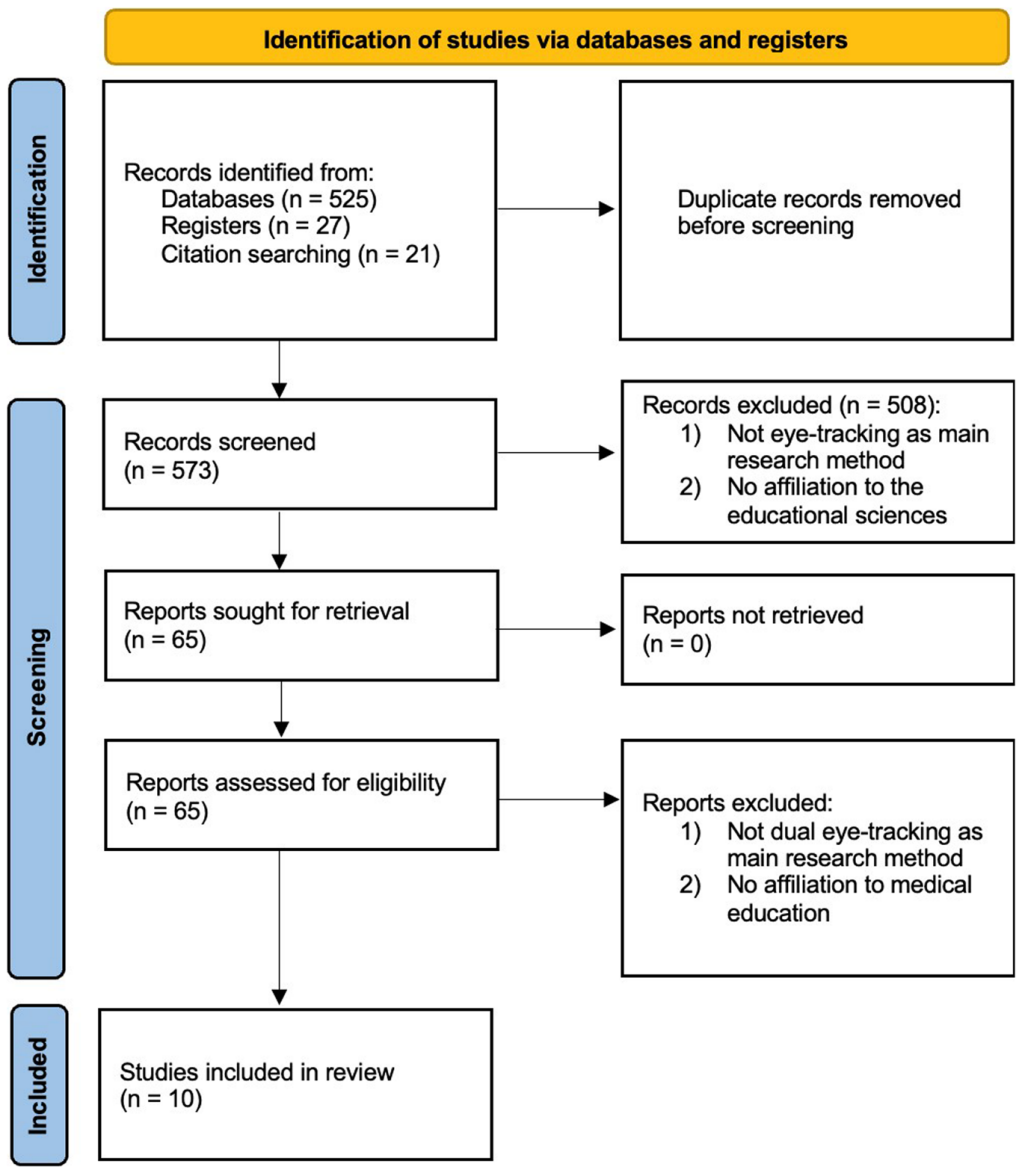
Work-flow for the scoping review.

Two researchers independently conducted a scoping literature review following the recommendations of the PRISMA-ScR guidelines ( Tricco *et al.*, 2018) including an identification, screening and inclusion step ( Figure 2). The full checklist is available under https://doi.org/10.6084/m9.figshare.26711728.v1. The review protocol has been registered at the Open Science Framework (OSF) under the reference ‘ugfhe’.

### Inclusion and exclusion criteria

We defined two main inclusion criteria: (1) the use of dual eye-tracking as the primary research method, and (2) a research focus on medical education, encompassing all stages from entry-level training to continuing education. These criteria were chosen to guarantee that dual eye-tracking was an integral part of the selected studies and to specifically target research in the field of medical education. We excluded gray literature. Discrepancies were resolved through consensus.

### Search strategy

First, a set of possible search-terms such as "dual," "dyadic," and "multiparty" eye-tracking were selected. These were combined into various search-strings, which were then piloted to assess their effectiveness. A focus was to specifically target DMET and DUET articles in medical education. The final set of search strings included ("dual” OR “dyadic” OR “multiparty” OR “eye tracking," OR "dual mobile eye tracking," OR "dual eye tracking", OR “dual eye tracking” OR “collaborative eye tracking” OR “tandem eye tracking” OR “paired eye tracking”) AND ("medical education" OR “medicine”).

Using these refined search strings, we screened multiple databases including PubMed, Education Resources Information Center (ERIC), APA PsycArticles, and Google Scholar. For cross-validation, we also examined five popular journals related to medical education: Academic Medicine, Medical Education, Advances in Health Sciences Education, Medical Teacher, and BMC Medical Education. Additionally, the references included in the articles that came up in the above screening were manually assessed to recover additional relevant articles. In a third step, we screened the publication lists of the authors of those recovered articles in the steps above. For this, the citation-based literature mapping tool ‘ResearchRabbit’ was used. The final set of studies was cross-validated with an expert in the field of dual eye-tracking methodology.

The last inclusion date was May 2024, culminating in *N* = 573 publications after removing duplicates ( Figure 2). The publications were further processed in EndNote X9 software.

### Study selection

Study selection followed a two-stage process, from coarse to fine selection. Initially, we included all types of studies, such as empirical research, conference reports, technical reports, and dissertations. This resulted in *n*=65 articles, which were fully reviewed. The full texts were then manually evaluated against the two primary inclusion criteria. This process yielded a final set of *n*=10 articles meeting all criteria.

### Data extraction and synthesis

Two researchers independently extracted data from the ten selected articles using a standardized table developed for this review in Excel® ( Table 1). The form captured information including authors, publication year, country in which the study was conducted, sample size, prior knowledge of participants (novice, intermediate, experts or mixed), nature of collaboration (teacher-teacher or student-student, teacher-student), type of dual eye-tracking (DUET or DMET), eye-tracking hardware and calibration, summary (primary objectives), area of application, metrics, and analytical strategies. Particular attention was given to the implications of the findings for medical training and practice.

**Table 1.** Summary of the characteristics of the included dual eye-tracking studies ( *n* = 10).

												Dual eye-tracking metrics			Analytical strategy
Author	Year	Country	Participants	Prior knowledge	Nature of interaction	Type	ET Hardware	ET Calibration	Study design	Summary	Area of Application	Gaze overlap/ JVA	Gaze Latency	Joint Mental Effort	Cross Recurrence
Chetwood *et al.*	2012	UK	28 subjects	Mixed: 7 surgeons and 23 non-clinicians	Asymmetric: supervisor with subject (all participants were subjects)	DUET	Two Tobii eye trackers (not specified)	Nine-point calibration	One-group repeated measures	This study projected a supervisor´s gaze onto the subject´s screen. They found improvements in time to complete tasks, eye-gaze latency, and number of errors.	Laparoscopic surgery	X	X	-	-
Kwok *et al.*	2012	UK	40 subjects	Mixed: 14 students, 13 fellows, 13 surgeons	Asymmetric: “master” with “assistant” (all participants were assistants)	DUET	Two Tobii X50	Calibration reported but not specified	Two-groups experimental	This study projected both operators' gazes into the screens. They found improvements in completion time, number of nodules extracted, distance traveled by the instrument, gaze latency, and gaze convergence.	Laparoscopic surgery	X	X	-	
Khan *et al.*	2012	Canada	4 experts, unclear number of residents	Mixed: Expert surgeons and residents	Not applicable	DUET	Tobii X50	Calibration reported but not specified	One-group observational	This study showed the gaze pattern of an expert to either an expert and novice. They found higher gaze overlaps in expert-expert dyads than in expert-novice dyads.	Laparoscopic surgery	X	-	-	-
Hajari *et al.*	2016	Canada	17 subjects forming 22 dyads	Mixed: surgical and non-surgical students	Symmetric	DUET	Tobii 1750 and Tobii X50	Not reported	One-group observational	This study evaluated metrics to distinguish between good and bad-performing dyads. They found that high-performing dyads showed lower gaze latency, and higher gaze overlap.	Laparoscopic surgery	X	X	-	X
He *et al.*	2016	Canada	22 dyads	Mixed: 14 international students, surgeons, and students forming 22 dyads	Symmetric	DUET	Tobii 1750 and Tobii X50	Not reported	Three-groups observational	This study assessed the frequency of movement de-synchronization between a surgeon and an assistant. They found that high-performing dyads showed fewer de-synchronizations than low-performing dyads.	Laparoscopic surgery	-	-	-	-
Zheng *et al.*	2016	Canada	14 subjects forming 22 dyads	Low: students	Symmetric	DUET	Tobii 1750 and Tobii X50	Not reported	One-group observational	This study assessed the relationship between gaze pattern and task performances. They found correlations between task time, de-synchronization events, and joint visual attention.	Laparoscopic surgery	X	X	-	X
Puckett	2016	USA	2 subjects forming 1 dyad	Mixed: 1 expert surgeon and 1 surgical resident	Asymmetric: expert and “novice”	DMET	Two EyeGuide Mobile Trackers	Not reported	Descriptive	This study presents a technical report to test the feasibility of DMET during a cholecystectomy operation.	Laparoscopic surgery	-	-	-	-
Hajari *et al.*	2018	Canada	17 subjects forming 22 dyads	Low: university students, office staff, visiting scholars without experience	Asymmetric	DUET	Tobii 1750 and Tobii X50	Not reported	One-group observational	This study evaluated metrics to distinguish between good and bad-performing dyads. They found good-performing dyads to have higher joint visual attention and lower gaze delay than bad-performing dyads.	Laparoscopic surgery	X	X	-	X
He	2019	Canada	Not applicable	Not applicable	Not applicable	DUET	Not applicable	Not applicable	Not applicable	Dissertation	Laparoscopic surgery	-	-	-	-
He *et al.*	2021	Canada	14 subjects forming 22 dyads	Mixed: 8 surgeons, 4 students	Asymmetric: surgeon and assistant	DUET	Tobii 1750 and Tobii X50	Not reported	One-group observational	This study assessed the pupil dilations of dyads during a coordination task as a surrogate for performance. They found that high-performing dyads have greater pupil size similarity than low-performing dyads, indicative of synchronization.	Laparoscopic surgery	-	-	X	-

Abbreviations: ET = eye-tracking, JVA = joint visual attention, DUET = dual (screen-based) eye-tracking, DMET = dual mobile eye-tracking

## Results

### Research on DUET and DMET in medical education is sparse

Our final review included ten peer-reviewed studies: two conference reports, one dissertation, six original research reports, and one technical report. The first dual eye-tracking study in medical education was conducted in 2012, and has been cited 121 times to date ( Chetwood *et al.*, 2012). All studies were conducted in the English-speaking countries Canada (70%), UK (20%), and the US (10%), and published between 2012 and 2021 ( Table 1). DUET was applied by 90% of the studies, while DMET comprised 10%. The terminology used to indicate dual eye-tracking technology is inconsistent, including terms like “collaborative eye tracking” ( Chetwood *et al.*, 2012), “tandem visual tracking” ( Puckett & Baronia, 2016), or not labeling the methodology specifically ( He *et al.*, 2021). An experimental design was found in two studies (20%), while observational approaches dominated the field (80%).

**Figure 3.**
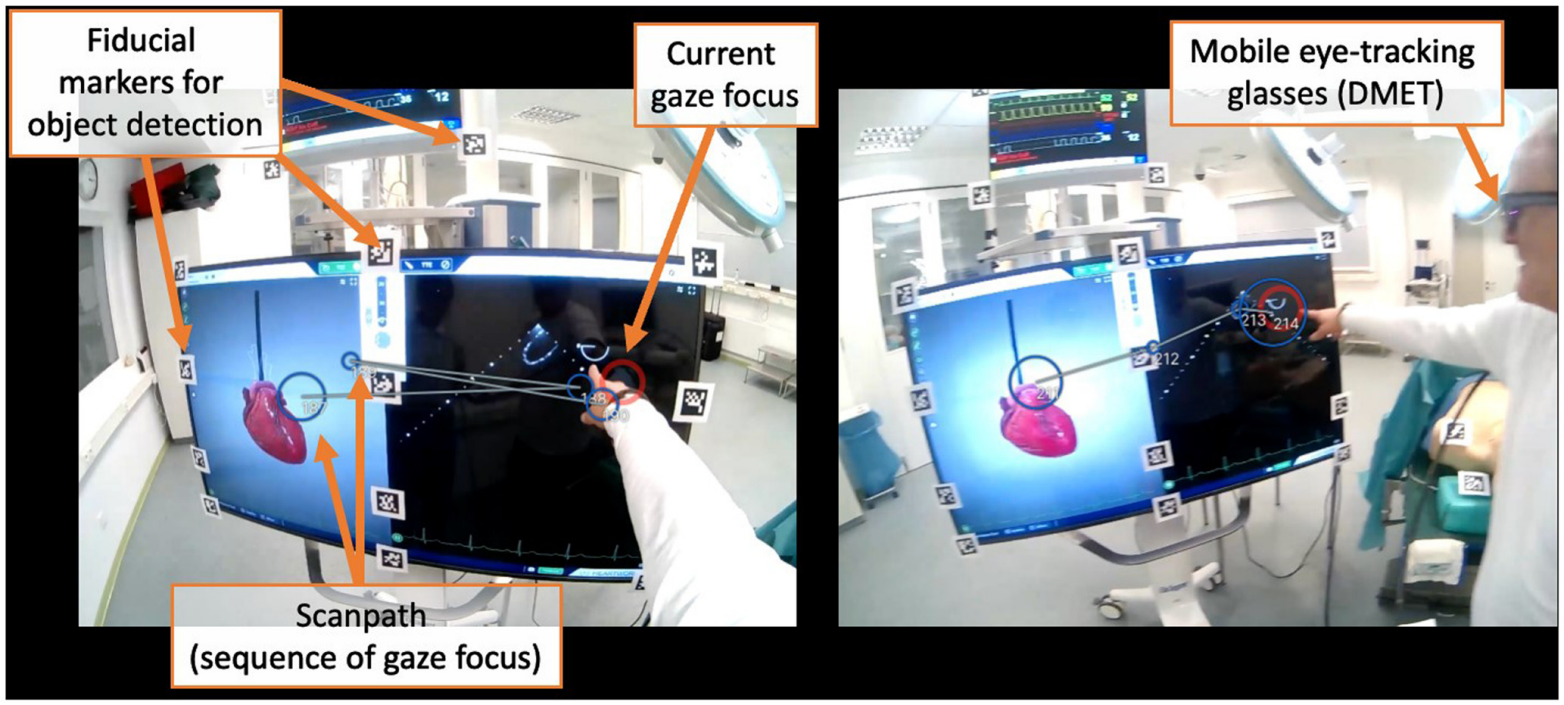
Example of a DMET setup during ultrasound training in an operating room. In this scenario, both the teacher and the student are wearing mobile eye-tracking glasses to track their eye movements. The image showcases the respective gaze focus of both the teacher (left) and the student (right) from their individual perspectives (red circle). At this particular moment, both are observing the ultrasound image displayed on the right side of the monitor, initiated by the teacher via finger pointing. The scanpath (the sequence of gaze focus) can be visualized afterward, which allows for detailed analysis of gaze patterns over time. The QR codes visible in the scene serve a crucial purpose in automated object detection. They enable the system to distinguish between different areas of interest, such as whether the gaze is focused on the anatomical image or the ultrasound image. *Note. These pictures were generated by the study authors.*

All studies were conducted within the context of surgical training, specifically focusing on laparoscopic surgery, with most of them utilizing a laparoscopic surgery simulator. One particular study (10%) evaluated DMET during an actual laparoscopic cholecystectomy surgery ( Puckett & Baronia, 2016). None of these studies were published in journals primarily dedicated to medical education. Instead, they appeared in surgery-specific, or interdisciplinary journals. An analysis of co-authorships reveals that the articles can be clustered into three different research teams. In all the articles, some authors' affiliations with surgical disciplines are evident. An example of a DMET setup is shown in Figure 3.

### Research questions that have been addressed using DUET or DMET technology

***DUET as an educational intervention to improve collaboration.*** Chetwood *et al.* (2012) investigated whether visualizing a supervisor's eye movements would improve the accuracy of participants performing a laparoscopic simulation task. They recorded the eye movements of an expert surgeon using a screen-based eye tracker and projected these in real-time onto the screen of the participant, who was also being eye-tracked. The study revealed that this gaze visualization method led to reduced completion times, fewer errors, and decreased gaze latency in two participants (i.e., the time delay between the gaze shifts indicating who is leading the conversation and who is following). They also examined the effectiveness in a subpopulation of non-English-speaking participants and found that these individuals particularly benefited from the intervention.

In a similar approach, Kwok *et al.* (2012) asked whether the gaze visualization of both collaboration partners (i.e., teacher seeing eye movements of the assistant and vice versa) during task-completion would enhance collaboration in a multi-robot surgical environment. They found that this intervention reduced the completion time, gaze latency, and the performance in the laparoscopic simulation. The DUET data were further corroborated by sensor data from the laparoscopic simulation, including metrics such as total distance traveled by the instrument, time required to complete the task, instrument tip distance traveled, and speed of instrument movement.

***DUET as a diagnostic tool to find gaze pattern that indicate successful collaboration.*** Khan *et al.* (2012) used an asynchronous DUET setup, where they recorded the eye movements of experts during cholecystectomy operations. They showed these recordings as “modeling examples” to either expert surgeons or resident surgeons and found that the gaze overlap (i.e., the percentage of time both participants have fixated on the same target throughout the whole task) was higher in expert-expert than in expert-novice conditions.

Hajari *et al.* conducted two studies with DUET in 2016 and 2018 with the goal of finding gaze patterns during surgical simulations that were associated with good and bad team performances. In Hajari *et al.* (2016), the dyads performed four different subtasks which required coordination and fine motor skills. During these, one participant had to maneuver the laparoscope, while the other moved the camera. In high-performing dyads (based on task times), they found higher levels of gaze overlap, as well as a decreased gaze latency. The results were interpreted within the theoretical framework of team cognition, which posits that team members contribute to, share, and build upon each other's knowledge, understanding, and mental models towards a common goal ( Fernandez *et al.*, 2017).

**Figure 4.**
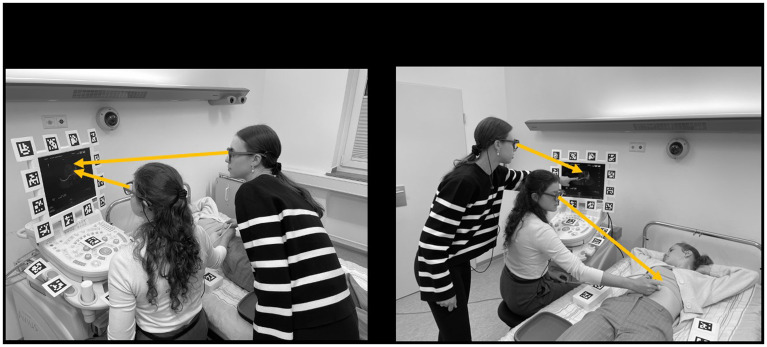
The image illustrates two contrasting events in a DMET setup. The left panel depicts a moment of synchronized visual focus between the two individuals (specifically, *joint visual attention*). Here, both participants are simultaneously directing their attention to the ultrasound monitor. This synchronized gaze behavior can be interpreted as a measure of coordination or alignment between the participants, indicating shared attention and potentially shared understanding or communication. In contrast, the scenario on the right panel shows a lack of visual synchronization. The participants are fixating on different objects or areas, indicating divergent attention. This misalignment of gaze could suggest differences in focus, understanding, or priorities between the individuals. *Note. These pictures were generated by the study authors.*

In a similar study setup, Zheng *et al.* (2016) and Hajari *et al.* (2018) confirmed these results. Besides gaze overlap and gaze delay as in the previous study, both studies additionally assessed joint visual attention (i.e., the percentage of time both participants fixated on the same targets *at the same time*), which they found to be higher in high-performing dyads. Furthermore, they introduced the metric of *de*-synchronization to record the number of events where the area of interest (i.e. target object) was out of sight. An example of joint visual attention is shown in Figure 4.

Using the same setup, He *et al.* (2021) analyzed the pupil-size similarity (i.e., the percentage of time both participants had the same pupil sizes, also known as *joint mental effort*) during a laparoscopic simulation as a surrogate for team performance. They found that pupil sizes were more alike and, thus “synchronized” in high-performing dyads. In addition, they analyzed the pupil size variation (i.e., the standard deviation of an individual’s pupil size data), and found lower pupil differences between team members in high-performing dyads when compared with low-performing dyads.

Some of these studies were critically assessed in Wenjing He's dissertation, published in 2019. In this work, she critiques the currently used assessment methods of teamwork in surgical education, such as observation-based or self-report measures because they are non-continuous and too slow to detect the fast nature of social interactions. Additionally, the dissertation highlights the technical challenges of conducting a DUET research project.

***Application of DMET during live surgery.*** One study used DMET in a medical education context. Puckett & Baronia (2016) used head-mounted mobile eye-trackers on a chief and resident surgeon together performing a real laparoscopic cholecystectomy operation. The authors suggest that novice surgeons can enhance their skills by studying the eye movements of the experts and comparing them with their own. In a technical report, the authors claim the methodology to be challenging but successful, and report no adverse effects. They further assessed fixations, saccades and pupil size of both surgeons, however without providing further empirical data. No follow-up studies from this group were found.

### Characteristics of DUET and DMET studies in medical education

Empirical studies included in this review (excluding the technical report and dissertation) used sample sizes ranging from 14–40 subjects per study, while none of the studies reported a power analysis. In Chetwood *et al.* (2012), participants formed multiple dyads resulting in a higher number of dyads than theoretically possible when testing each participant only once.

Most of the studies (70%) included participants with mixed prior knowledge often pairing experts with novices, and one study (10%) tested participants with no prior knowledge. Regarding the nature of interaction, 40% of the them were asymmetric interactions, i.e. expert-novice-dyads, and 30% were symmetric, i.e. novice-novice-dyads.

All studies used commercial eye-tracking devices. The majority used the same setup, which consisted of two screen-based eye trackers Tobii X50 and Tobii 1750. One study (10%) combined two Tobii X50 devices and the DMET study used two EyeGuide Mobile Trackers. Synchronization of the data streams was achieved either manually or using the software *Labview* with a custom script. One study (10%) reported self-assessed quality measures, i.e. precision or accuracy of the eye trackers, while most of the studies (90%) referred to the specifications provided by the manufacturers.

## Discussion

This review is the first to survey the use of dual eye-tracking technology in the context of medical education. Our initial screening revealed a significant increase in publications on eye-tracking in medical education, yet a notable gap in the literature specifically addressing DUET and DMET exists. Here, we identified just ten studies, using dual eye-tracking methodology in medical education setting, which do report a great potential to provide rich, dynamic data on social interactions and collaborative learning processes. Thus, the low number of studies clearly argues that much of the potential is actually unused. This is in contrast to higher educational research, where a recent systematic review found 59 publications that have used dual eye tracking methodologies to study joint visual attention ( Schneider, under review).

### Application of dual eye-tracking in medical education

Our review identified two primary applications of DUET in medical education. The first set of studies employed DUET as an educational *intervention* to improve teamwork through shared gaze visualization ( Chetwood *et al.*, 2012; Kwok *et al.*, 2012). By visualizing where team members are looking, DUET facilitated coordination and communication among team members. Specifically, visualizing eye movements helped team members anticipate each other's actions in real-time, thereby coordinating their efforts, and enhancing overall team performance and learning outcomes. This strand of application is promising and builds on related work from single eye-tracking literature, such as eye movement modeling examples ( Darici *et al.*, 2023a; Gegenfurtner *et al.*, 2017), or gaze cueing. These studies show that learner’s profit from seeing an expert’s eye movements, which guides attention and thought processes, helping them focus on task-relevant information.

The other set of studies used DUET as a *diagnostic tool* to assess the quality of social interactions ( Hajari *et al.*, 2016; He & Zheng, 2016 and He, 2019; He *et al.*, 2021; Khan *et al.*, 2012; Zheng *et al.*, 2016). Researchers have identified several collaborative metrics, frequently focusing on gaze overlap and joint visual attention, as indicators of attention and cognition in dyadic interactions. These metrics expand the range of eye-tracking metrics in SIET research, and provide a new approach to address group-level research questions.

### Limitations of the current literature

Several aspects limit the impact of the current literature. First, the studies use different terminology to refer to dual eye-tracking. This variability in terminology not only makes it difficult for researchers to communicate effectively but also poses challenges for meta-analyses and systematic reviews. It can lead to relevant studies being overlooked or misinterpreted. Establishing a standardized vocabulary for dual eye tracking research, particularly in the context of medical education, would greatly benefit the field's development and integration into broader research and practice.

Second, although some studies reference theoretical models, such as team cognition ( Hajari *et al.*, 2016 and Hajari *et al.*, 2018; Zheng *et al.*, 2016), and shared cognition ( He *et al.*, 2021) the theoretical foundation should be expanded. For example, Kirschner *et al*. (2009) discussed an approach, where collaborative learning can be understood in terms of how it affects individual and group cognitive load.

Third, many studies were conducted exclusively in surgical settings concentrating on collaboration during laparoscopic procedures. This narrow focus limits the generalizability of findings to other medical specialties and educational contexts.

Fourth, the studies predominantly use screen-based DUET techniques, which may not fully capture the complexity of real-world medical interactions. Thus, there is a need for research employing DMET to investigate more realistic scenarios beyond screen-based applications.

Fifth, all studies were published outside of medical education journals. This reduces their visibility within the medical education community and may result in infrequent citations by researchers in this field.

Sixth, the involvement of only three independent research groups in these studies underscores the need for broader and more diverse research efforts to validate and extend these initial findings.

**Figure 5.**
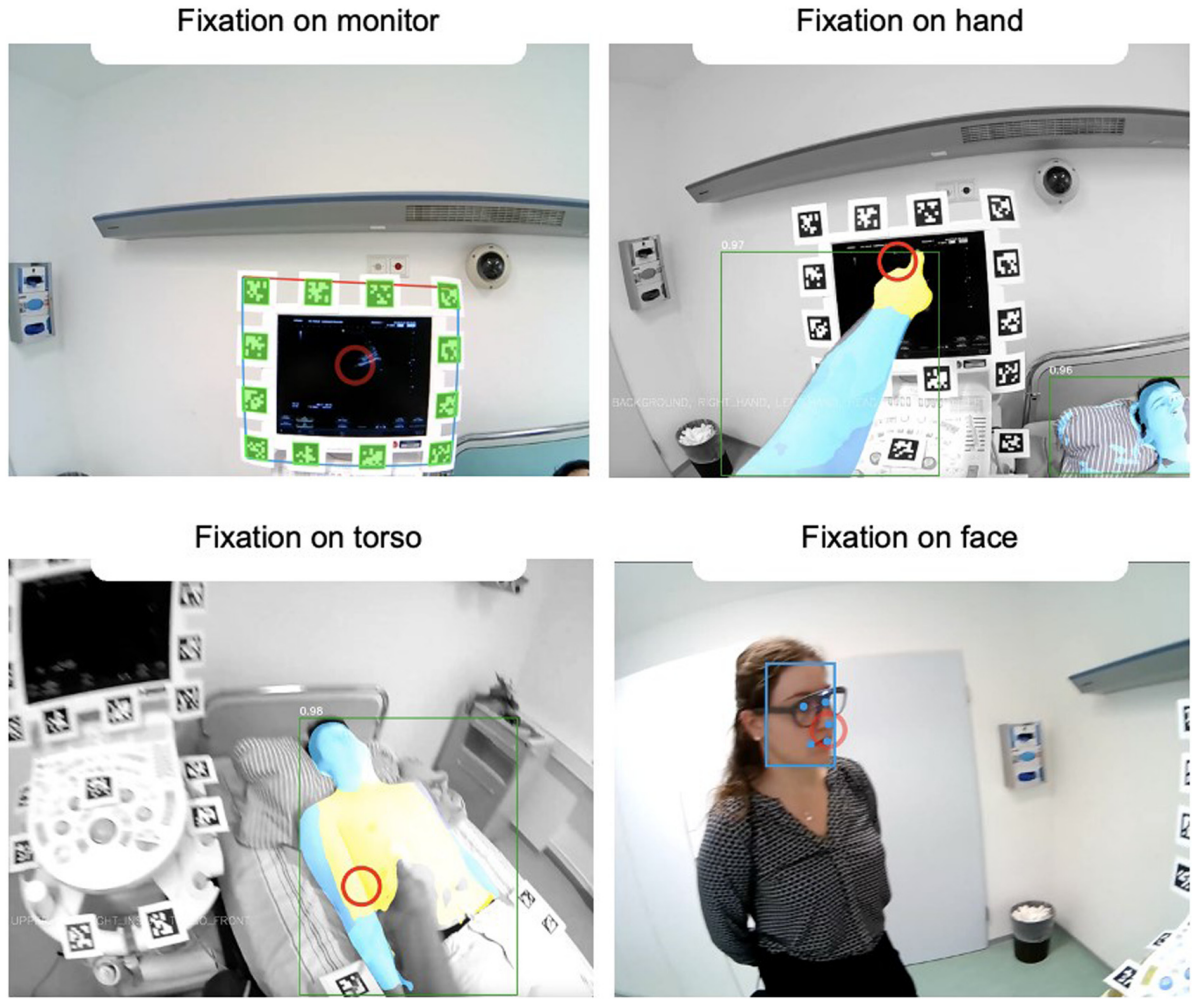
The figure shows a dual eye-tracking setup from a participant's perspective. The red circle represents the current gaze focus. In DMET settings, determining which areas of interest are being fixated is more challenging than in screen-based methods due to varying positioning and movement in space having an impact on the target size. However, various technical approaches make it possible to quantify and automate this process, allowing for analysis of long recordings. For example, panel A demonstrates how fixation on the monitor is captured using fiducial markers (i.e., QR codes), a strategy referred to as *marker mapping*. Panels B-D showcase the use of different DensePose analyses, based on computer vision techniques, to capture fixations on various body parts. As this field is rapidly advancing, significant improvements and simplifications are expected in the future. *Note. These pictures were generated by the study authors.*

Finally, most of the studies were conducted and published in 2016 or earlier, utilizing eye-tracking technology that is considered outdated by current standards ( Figure 5). This technological limitation may impact the applicability of their results to present-day medical education and practice.

### Opportunities for future dual eye tracking research in medical education

The following suggestions aim to foster use of dual eye-tracking studies in medical education.

***Expanding application beyond surgery.*** While dual eye-tracking has shown promise in surgical settings, its potential extends to other areas of medical practice. This broad applicability becomes evident when considering the potential to study social interactions. For example, the CanMEDS roles that define a medical expert—Communicator, Collaborator, Leader, Professional, Scholar, and Health Advocate ( Ringsted *et al.*, 2006), inherently involve social interaction suggesting that dual eye-tracking could be a valuable tool across the spectrum of medical education.

Social interactions are based on person-person interactions. Those are guided by small cues many of which are produced by eye-movements ( Pfeiffer *et al.*, 2013). Thus tracking those will provide metrics to quantify social interactions. As a sensor-based methodology, dual eye-tracking can capture these swift gaze sequences, making nuanced measurements possible ( Schneider *et al.*, 2021). Its high temporal resolution might be particularly valuable for examining rapid processes like attention focus and eye contact. Laparoscopic surgery is a fitting example, necessitating collaboration between at least two surgeons with live coordination and leadership-follower dynamics. Similarly, emergency situations are characterized by rapid social gaze sequences ( Tekïn *et al.*, 2023). In these contexts, dual eye-tracking could offer insights into dyad’s real-time decision-making and attentional focus during critical procedures. Conversely, it could help evaluate potential barriers to successful action and be used for increase the quality of debriefing sessions.

Expertise research shows that gaze behavior changes as a function of experience ( Darici *et al.*, 2024). Dual eye-tracking now enables the study and improvement of social interactions across various experience levels, such as those occurring in interdisciplinary consultations between two professions. This methodology could enhance medical education by examining teacher-student interactions during cognitive apprenticeship situations, like sonography training. It also presents a new perspective for studying interprofessional teamwork by revealing which areas of interest dyads focus on most frequently and correlating these findings with dyad performance measures.

Lastly, the mental health field could benefit from dual eye-tracking's ability to analyze therapist-client communication patterns, particularly by collecting metrics on *social gaze* ( Pfeiffer *et al.*, 2013).

Future research in dual eye tracking could be expanded to encompass multiple participants through *multiparty eye-tracking* ( Reuscher *et al.*, 2023). This approach would allow for the investigation of visual attention patterns within larger groups, potentially revealing complex interaction dynamics during high-stakes scenarios, such as resuscitation efforts or the collaborative decision-making processes that occur during clinical ward rounds. They could be used to understand communications errors ( Gundrosen *et al.*, 2016), which reduce patient safety.

***Advancing metrics beyond gaze alignment.*** We found that dual eye-tracking metrics emphasize various forms of gaze alignment. This emphasis likely originates from the rich body of research on joint visual attention (JVA), a concept with deep roots in developmental and clinical psychology ( Siposova & Carpenter, 2019). JVA is considered crucial for language acquisition in children, and difficulties in establishing eye contact or gaze-cued JVA in social contexts have been associated with autism spectrum disorders. While these studies have traditionally centered on child development, recent research has begun to explore JVA's relevance in higher education settings ( Schneider, under review). For example, studies by Richardson and Dale (2005) examined how conversational partners achieve synchrony in their gaze patterns, finding that higher levels of gaze coordination were associated with better communication outcomes. Neider *et al.* (2010) expanded this work by investigating gaze behavior in more complex collaborative tasks, such as problem-solving scenarios. Their findings indicated that successful task performance was often preceded by periods of highly synchronized gaze, suggesting that visual attention plays a crucial role in coordinating actions and achieving goals. This expansion of focus suggests that the principles of JVA, as measured through dual eye-tracking, may have broader applications in understanding and enhancing adult learning and collaboration in professional contexts such as medical education.

However, dual eye tracking offers more metrics that can be used to understand social interactions. For example, future studies could compute metrics that capture leadership behaviors, and cycles of collaboration ( Table 2) that have been used in non-medical research fields ( Schneider, under review). For example, tracking how gaze patterns indicate leadership roles or how gaze synchrony evolves during different phases of a collaborative task could provide richer data on team dynamics. By triangulating self-reports and observational studies with dual eye-tracking metrics, it may provide a more comprehensive picture of social interactions ( Walsh, 2013).

**Table 2.** Popular gaze metrics for dual eye tracking research.

Dual eye tracking metrics	Description	Practical application	Reference
Gaze overlap	The extent to which two participants focus on the same area during a collaborative task. These focal points may result from visually striking features (bottom-up processing) or reflect purposeful focus based on knowledge and goals (top-down processing).	Gaze overlap reveals areas that attract observers' attention. This metric helps understand how people perceive and prioritize visual information.	Zheng *et al.*, 2016
Joint visual attention	Moments two participants *simultaneously* focus on the same area. It typically happens when one individual indicates an object, often by pointing, and the other person shifts their gaze to observe the same object.	Joint visual attention is a temporal extension of gaze overlap. It can be used to understand how people coordinate and synchronize their visual behavior over time.	Schneider *et al.*, 2021
Gaze delay	The time lag between the gaze shifts of two participants. It measures the temporal delay in visual attention shifts, reflecting how quickly one participant’s gaze follows or reacts to the gaze shift of the other participant.	Gaze delay is used to determine leader-follower dynamics. It indicates who initiates shifts in attention and who responds, providing insights into the natural roles and influence patterns within interactions.	Hemshorn de Sanchez *et al.*, 2022
Joint mental effort	The degree of shared pupil dilation between participants. Since pupil size can reflect cognitive load, similar changes in pupil size can indicate synchronized cognitive effort.	Joint mental effort offers insights into moments of mutual engagement or coordinated cognitive processing during interactions.	Sharma & Olsen, 2022
Mutual gaze	The situation where two participants look directly into each other’s eyes.	Mutual gaze is a significant aspect of non-verbal communication, often indicating engagement, attention, and connection between individuals. Analyzing the frequency and duration of mutual gaze provides insights into social dynamics and the quality of interaction between individuals.	Pfeiffer *et al.*, 2013
Reciprocal gaze	A back-and-forth exchange of gaze between participants. It captures the dynamic process of one person looking at another, who then responds by looking back.	Gaze reciprocity patterns reveal the fluidity of nonverbal communication and the level of mutual engagement in an interaction.	Pfeiffer *et al.*, 2013
Gaze aversion	The intentional act of diverting one's gaze away from another person or object.	Gaze aversion serves various cognitive, social, and emotional functions. For example, it regulates intimacy, and can communicate respect or submission in certain cultures. Emotionally, it helps manage discomfort.	Pfeiffer *et al.*, 2013

Compared to traditional screen-based eye-tracking, DMET provides unique applications, especially in its ability to capture 'social gaze' patterns during real-world interactions (e.g., Mayrand *et al.*, 2023). Wearable devices facilitate the analysis of gaze behavior on moving objects in a room, allowing researchers to study how individuals track dynamic elements in their environment and interact socially. Thereby, DMET can measure the reciprocal nature of eye contact between participants, providing insights into the dynamics of face-to-face communication ( *mutual gaze*). For example, Rogers *et al.* (2018) used a DMET approach to analyze gaze patterns during real-world conversations, and found consistent differences in which part of the face is fixated by individuals. This capability has led to the development of metrics such as *reciprocal gaze*, which examines how one participant's gaze influences the other's gaze behavior, or *gaze aversion*, which assesses instances where participants deliberately avoid eye contact during social interactions ( Pfeiffer *et al.*, 2013).

## Limitations

This scoping review on dual eye-tracking in medical education faced several limitations. The small number of identified studies and their narrow focus on surgical settings, particularly laparoscopic procedures, limit the generalizability of findings. These limitations underscore the need for more diverse, comprehensive, and up-to-date research to fully explore the potential of dual eye-tracking in medical education.

## Conclusion

Social interactions in medical education promote the growth of proficient, empathetic, and collaborative healthcare professionals ( Darici *et al.*, 2022). Through clinical rotations and simulation labs, students engage in team-based problem solving and critical patient care scenarios; through mentorship, novice practitioners learn directly from experienced physicians, observing and participating in patient care. Because social interactions are the foundation of effective medical education, there is a need to develop better tools to capture and support interactions between students and teachers. This scoping literature review shows that dual eye-tracking is an innovative and potentially transformative methodology that can positively impact social interactions. However, it is not widely used yet, and there are few studies on its use in medicine. This situation motivates the need for more research to explore and take advantage of how dual eye-tracking can benefit medical learning.

## Data Availability

No data are associated with this article. ***Reporting guidelines.*** Figshare: PRISMA-ScR checklist for 'Looking at social interactions in medical education with dual eye-tracking technology: A scoping review'. https://doi.org/10.6084/m9.figshare.26711728 ( Tricco *et al.*, 2018) Data are available under the terms of the Creative Commons Attribution 4.0 International license (CC-BY 4.0).

## Biographical notes

Johannes Lorenz is a final year medical student

Juliane Zevano is a third-year medical student

Nils Otto, PhD is a postdoctoral researcher

Bertrand Schneider, PhD is an associate professor

Markus Missler, MD is a full professor

Dogus Darici, MD, MME, MSc. is a postdoctoral researcher
