# Supplementary material for: Looking at Social Interactions in Medical Education with Dual Eye-Tracking Technology: A Scoping Review
Source: MedEdPublish (2016). 2024 Dec 18;14:215. Originally published 2024 Oct 10. [Version 2] doi: 10.12688/mep.20577.2 (PMC11809160; doi:10.12688/mep.20577.2)
Supplement: Peer review history

# Reviewer report: Reviewer response for version 2

**DOI:** 10.21956/mep.22317.r40816

**Published:** 2025-01-23

**Authors:** Irene González-Díez

**Affiliations:** 1 University of Burgos, Burgos, Castile and León, Spain

I believe that the topic addressed by the authors is relevant to the daily practice of education in the medical and healthcare fields in general. In this regard, I consider the review to be focused and clear in its objectives and conclusions, while also providing new insights that enable the improvement of teaching practices.

Are the rationale for, and objectives of, the Systematic Review clearly stated?

Yes

Is the statistical analysis and its interpretation appropriate?

Not applicable

Are sufficient details of the methods and analysis provided to allow replication by others?

Yes

Are the conclusions drawn adequately supported by the results presented in the review?

Yes

Reviewer Expertise:

Educational Psychology, Health Sciences, Eye Tracking

I confirm that I have read this submission and believe that I have an appropriate level of expertise to confirm that it is of an acceptable scientific standard.

# Reviewer report: Reviewer response for version 2

**DOI:** 10.21956/mep.22317.r40631

**Published:** 2024-12-30

**Authors:** Lina Kaminskiene

**Affiliations:** 1 Vytautas Magnus University, Kaunas, Lithuania

The paper is well-developed, and I have only minor comments which may contribute to the improvement of the paper.

1. The authors indicate that in 80% of studies observational approaches dominated, it would be useful to include more specific information how the data were reported, providing specific statistical or qualitative data. To make my comment clear, I give one example. The authors write: “ *Besides gaze overlap and gaze delay as in the previous study, both studies additionally assessed joint visual attention (i.e., the percentage of time both participants fixated on the same targets at the same time), which they found to be higher in high-performing dyads.*” It would be useful to know what the percentage was here.

A comment regarding a smaller number of studies where DUET was employed as an educational intervention would be also interesting.

Are the rationale for, and objectives of, the Systematic Review clearly stated?

Yes

Is the statistical analysis and its interpretation appropriate?

Not applicable

Are sufficient details of the methods and analysis provided to allow replication by others?

Yes

Are the conclusions drawn adequately supported by the results presented in the review?

Yes

Reviewer Expertise:

Educational Sciences

I confirm that I have read this submission and believe that I have an appropriate level of expertise to confirm that it is of an acceptable scientific standard, however I have significant reservations, as outlined above.

# Reviewer report: Reviewer response for version 2

**DOI:** 10.21956/mep.22317.r40588

**Published:** 2024-12-21

**Authors:** Bin Zheng

**Affiliations:** 1 University of Alberta, Edmonton, Canada

I have no additional comments.

Are the rationale for, and objectives of, the Systematic Review clearly stated?

Yes

Is the statistical analysis and its interpretation appropriate?

Yes

Are sufficient details of the methods and analysis provided to allow replication by others?

Yes

Are the conclusions drawn adequately supported by the results presented in the review?

Yes

Reviewer Expertise:

Surgical education, surgical team training, performance assessment, skill acquisition, surgical simulation, ergonomic assessment in surgical setting

I confirm that I have read this submission and believe that I have an appropriate level of expertise to confirm that it is of an acceptable scientific standard.

# Reviewer report: Reviewer response for version 1

**DOI:** 10.21956/mep.22024.r39619

**Published:** 2024-11-06

**Authors:** Bin Zheng

**Affiliations:** 1 University of Alberta, Edmonton, Canada

As a literature review paper, the authors present a clear search strategy and well-defined criteria for paper selection. Tables and figures effectively visualize results from a pool of 10 selected studies. The discussion highlights the contributions of dual eye-tracking in identifying social interaction behaviors across various settings and explores its applications in medical education. The discussion around limitations in past research are reasonable and point to opportunities for future research.

Are the rationale for, and objectives of, the Systematic Review clearly stated?

Yes

Is the statistical analysis and its interpretation appropriate?

Yes

Are sufficient details of the methods and analysis provided to allow replication by others?

Yes

Are the conclusions drawn adequately supported by the results presented in the review?

Yes

Reviewer Expertise:

Surgical education, surgical team training, performance assessment, skill acquisition, surgical simulation, ergonomic assessment in surgical setting

I confirm that I have read this submission and believe that I have an appropriate level of expertise to confirm that it is of an acceptable scientific standard.

# Reviewer report: Reviewer response for version 1

**DOI:** 10.21956/mep.22024.r39322

**Published:** 2024-10-15

**Authors:** Masahiro Sugimoto

**Affiliations:** 1 Keio University, Tsuruoka, Japan

Review of papers comparing the eye movements of supervisors and participants, well structured and generally well described.

 Eye movements have multiple roles, including those for gathering and perceiving information and those for decision-making. It should be added how the detailed meaning of these eye movements is analysed and interpreted in each study.

 As stated in ‘Characteristics of DUET and DMET studies in medical education’, what is the impact on eye gaze when participants have different or the same prior knowledge?

 ‘Application of DMET during live surgery.’ describes the study in actual surgery. Are there differences in eye movements between virtual and actual practice, such as simulations and post-operative videos?

 ResearchRabit and EndNote software should be given version, company, city and country information.

Are the rationale for, and objectives of, the Systematic Review clearly stated?

Yes

Is the statistical analysis and its interpretation appropriate?

Not applicable

Are sufficient details of the methods and analysis provided to allow replication by others?

Yes

Are the conclusions drawn adequately supported by the results presented in the review?

Yes

Reviewer Expertise:

Eye-tracking, bioinformatics

I confirm that I have read this submission and believe that I have an appropriate level of expertise to confirm that it is of an acceptable scientific standard, however I have significant reservations, as outlined above.
